# RAGE ligands stimulate angiotensin II type I receptor (AT1) via RAGE/AT1 complex on the cell membrane

**DOI:** 10.1038/s41598-021-85312-4

**Published:** 2021-03-11

**Authors:** Serina Yokoyama, Tatsuo Kawai, Koichi Yamamoto, Huang Yibin, Hiroko Yamamoto, Akemi Kakino, Hikari Takeshita, Yoichi Nozato, Taku Fujimoto, Kazuhiro Hongyo, Toshimasa Takahashi, Futoshi Nakagami, Hiroshi Akasaka, Yoichi Takami, Yasushi Takeya, Ken Sugimoto, Tatsuya Sawamura, Hiromi Rakugi

**Affiliations:** 1 Department of Geriatric and General Medicine, Osaka University Graduate School of Medicine, 2-2 Yamadaoka, Suita, 565-0871 Japan; 2 Department of Molecular Pathophysiology, Shinshu University Graduate School of Medicine, Matsumoto, Nagano, 390-8621 Japan

**Keywords:** Molecular biology, Cardiology

## Abstract

The receptor for advanced glycation end-products (RAGE) and the G protein-coupled angiotensin II (AngII) type I receptor (AT1) play a central role in cardiovascular diseases. It was recently reported that RAGE modifies AngII-mediated AT1 activation via the membrane oligomeric complex of the two receptors. In this study, we investigated the presence of the different directional crosstalk in this phenomenon, that is, the RAGE/AT1 complex plays a role in the signal transduction pathway of RAGE ligands. We generated Chinese hamster ovary (CHO) cells stably expressing RAGE and AT1, mutated AT1, or AT2 receptor. The activation of two types of G protein α-subunit, Gq and Gi, was estimated through the accumulation of inositol monophosphate and the inhibition of forskolin-induced cAMP production, respectively. Rat kidney epithelial cells were used to assess RAGE ligand-induced cellular responses. We determined that RAGE ligands activated Gi, but not Gq, only in cells expressing RAGE and wildtype AT1. The activation was inhibited by an AT1 blocker (ARB) as well as a RAGE inhibitor. ARBs inhibited RAGE ligand-induced ERK phosphorylation, NF-κB activation, and epithelial–mesenchymal transition of rat renal epithelial cells. Our findings suggest that the activation of AT1 plays a central role in RAGE-mediated cellular responses and elucidate the role of a novel molecular mechanism in the development of cardiovascular diseases.

## Introduction

Advanced glycation end-products (AGEs) are glycated proteins or lipids produced by non-enzymatic reactions between proteins and sugars, and are upregulated under pathophysiological conditions such as hyperglycemia in diabetic patients^1,2^. The receptor for AGE (RAGE)—a type I transmembrane glycoprotein of the immunoglobulin superfamily—is a multiligand signal transduction receptor that can interact with multiple ligands, including AGEs, high-mobility group box-1 (HMGB1), and certain members of the S100/calgranulin family, to activate the ERK1/2 (p44/p42 MAP kinase) and JAK/STAT signaling pathways and promote downstream activation of NF-κB^3^. Previous studies have revealed that mammalian Diaphanous homolog 1 (mDia1)—a member of the formin family of Rho GTPase effector proteins— plays a key role in regulating RAGE-mediated intracellular signaling. The cytoplasmic domain of RAGE interacts with mDia1 to promote RAGE-mediated intracellular signaling and cellular migration^3–5^.

Angiotensin II (AngII) type I receptor (AT1)—a member of the G-protein coupled receptor (GPCR) family—in the renin–angiotensin system (RAS) plays an important role in the pathogenesis of cardiovascular diseases and diabetes^6^. Previous studies have revealed several novel mechanisms for AT1 activation, including heterodimeric formation with other GPCRs^7–11^ and biased activation mechanisms by allosteric ligands^12–14^. Interestingly, Pickering et al. reported that AT1 forms a heteromeric complex with RAGE, and that AngII-induced inflammation and atherogenesis are mediated by the transactivation of RAGE^15^. However, it remains unknown whether there is a different directional crosstalk in this complex, whereby AT1, independent of mDia-1, is involved in RAGE-induced cellular signaling. We previously demonstrated that AT1 forms a complex with the lectin-like oxidized LDL (oxLDL) receptors (LOX-1) on the cell membrane, whereby oxLDL activates AT1 and causes vascular endothelial dysfunction in mice^16^. Given these findings, we hypothesized that RAGE ligands can initiate cell signaling by interacting with the RAGE/AT1 complex on the cell membrane.

Herein, we investigated whether RAGE ligands can activate G proteins and their downstream signaling pathways in an AT1-dependent manner. We also investigated whether RAGE ligand-mediated cellular responses are abolished by the inhibition of AT1 in renal epithelial cells.

## Methods

### Cell culture

NRK52E cells (87,012,902, ECACC, UK) or transgenic CHO cells were cultured in DMEM (044–29,765, Wako, Japan) supplemented with 5% fetal bovine serum (FBS; Gibco, Thermo Fisher Scientific, USA) or F12 nutrient mixture with GlutaMAX-I (Thermo Fisher Scientific, USA) containing 10% FBS and appropriate selection reagents, respectively. Gene transcription in CHO cells was induced by adding 100 ng/mL doxycycline (Merck Millipore, USA) . All cells were serum-starved for 24 h before stimulation.

### Construction of plasmid vectors for stable transformants

The V5-tagged human RAGE Tet-On vector was constructed by serially subcloning full-length human RAGE into pcDNA6.2/V5/GW/D-TOPO (Thermo Fisher Scientific, USA) and pTRE2hyg (Clontech, USA) (pTREhyg-V5-hRAGE). The Tet-On human AT1 vector tagged with signal peptide-HA-FLAG at the N-terminus was created in a previous study (pTRE2hyg-HA-FLAG-hAT1)^16^. The Tet-On mutant AT1 vector was constructed using the PrimeSTAR mutagenesis basal kit (Takara, Japan) by deleting amino acids 221 and 222 from pTRE2hyg-HA-FLAG-hAT1 (pTRE2hyg-HA-FLAG-hAT1mt). The Tet-On human AT2 vector tagged with signal peptide-HA-FLAG at the N-terminus was constructed by subcloning full-length human AT2, according to the previous study (pTRE2hyg-HA-FLAG-hAT2)^16^. The Tet-On human mDiapahnous-1(mDia-1) was constructed by subcloning full-length human mDia-1 into pTRE2hyg (pTRE2hyg-hmDia-1).

### Stable transformants

CHO-RAGE cells were generated by the stable transfection of CHO-K1 Tet-On cells (Clontech, USA) with pTRE2hyg-V5-RAGE, according to a previous study^16^. CHO-RAGE-AT1, CHO-RAGE-AT1mt, and CHO-RAGE-AT2 were generated by the co-transfection of pTRE2hyg-HA-FLAG-hAT1, pTRE2hyg-HA-FLAG-hAT1mt, and pTRE2hyg-HA-FLAG-hAT2 and pSV2bsr vector into CHO-RAGE cells, respectively^16^. CHO-RAGE transfected with mDia-1 was also generated by the co-transfection of pTRE2hyg-hmDia-1 and pSV2bsr vector into CHO-RAGE cells. Stable transformants were selected with 400 μg/mL hygromycin B (Wako, Japan) and 10 μg/mL blasticidin S (Funakoshi, Japan). Resistant clones expressing the respective genes in response to doxycycline were selected for use in experiments.

### Immunofluorescence staining

Tagged RAGE and AT1 / AT1mt / AT2 in genetically engineered CHO cells were detected using mouse anti-V5 (Nacalai, Japan) and rat anti-FLAG (Novus Biologicals, USA) antibodies, in combination with rabbit Alexa488-conjugated anti-rat IgG and goat Alexa594-conjugated anti-mouse IgG (Thermo Fisher Scientific, USA), respectively. 4′,6-diamidino-2-phenylindole (DAPI) (Sigma-Aldrich, USA) was used to counterstain the nuclei. Images were acquired with a fluorescence microscope (BZ-X700, Keyence, Japan).

### Cell-based ELISA

Cells were seeded at a density of 150,000 cells per well onto 96-well transparent cell-culture plates and incubated overnight at 37 °C. The following day, the cultures were transferred to serum-free conditions and the cells were further incubated for 24 h. Thereafter, the cells were fixed by 4% paraformaldehyde without permeabilization, incubated with mouse anti-V5 or rat anti-FLAG antibodies, and then incubated with HRP-conjugated mouse or rat secondary antibodies, respectively. TMB reagents (SeraCare Life Sciences, USA) were then added to each well and the colorimetric reaction was neutralized using a stopping solution (SeraCare Life Sciences, USA). OD 450 values were measured using Multiskan Go (Thermo Fisher Scientific, USA). Measurement value of each sample was adjusted by subtracting that of negative control incubated with the corresponding secondary antibody in the absence of the first antibody.

### Co-immunoprecipitation assay

Membrane proteins were extracted using the transmembrane protein extraction kit (Merck Millipore, USA). Immunoprecipitation was performed using the anti-FLAG-M2 affinity gel (Sigma-Aldrich, USA). FLAG-fusion proteins were eluted with 3 × FLAG peptide (Sigma-Aldrich, USA). Subsequently, purified proteins were separated by SDS-PAGE under reducing or non-reducing conditions, transferred to PVDF membranes, and subjected to western blotting using antibodies against FLAG and V5.

### Western blotting

The cells were washed twice with PBS, and lysed using M-PER mammalian protein extraction reagent (Thermo Fisher Scientific, USA) with protease (Thermo Fisher Scientific, USA) and phosphatase (Nacalai Tesque, Japan) inhibitors. For western blot analysis, proteins were separated by SDS-PAGE and transferred onto PolyVinylidene DiFluoride (PVDF) membranes. The membranes were blocked with 5% non-fat dry milk and incubated overnight at 4 °C using the following primary antibodies: mouse V5 (Nacalai Tesque, USA), rat FLAG (Novus Biologicals, USA), rabbit ERK1/2, rabbit phospho-ERK1/2, mouse α-Tubulin (Cell Signaling Technology, USA), or mouse α smooth muscle actin (SMA) (Sigma-Aldrich, USA). Bands were detected using Chemi-Lumi One Super (Nacalai Tesque, Japan) and Chemiluminescence detection system LAS-4000 Mini (GE Healthcare, USA).

### In situ proximity ligation assay

The in situ proximity ligation assay (PLA) was conducted using the Duolink kit (Sigma-Aldrich, USA) to determine the proximity of membrane receptors using mouse anti-V5 antibody (Nacalai, Japan) and rabbit anti-FLAG antibody (MBL, Japan), as reported previously^16^. Images were acquired using the BZ-X700 fluorescence microscope. Quantitative fluorescence cell image analysis was performed using the BZ-X analyzer system (Keyence, Japan).

### Quantification of cellular IP1 accumulation

Cells were seeded at a density of 8000 cells per well onto 96-well transparent cell culture plates and incubated overnight at 37 °C. The following day, cultures were transferred to serum-free conditions and further incubated for 24 h. Thereafter, the cells were treated for 1 h with IP1 stimulation buffer mixed with the same amount of DMEM without phenol including vehicle, RAGE-BSA, HMGB-1, and angiotensin II at an indicated concentration at 37 °C, 5% CO2. Triton X was then added at a final concentration of 1%, and cell lysates were prepared after shaking the plates for 30 min. Finally, the cell lysates were transferred to 384-well white plates and IP1 levels were measured using the IP-One assay kit (Cisbio, France).The emission signals were measured at 620 and 665 nm after excitation at 320 nm, using the ARTEMIS plate reader(Furuno Electric Co. Ltd., Japan).

### Quantification of cellular cAMP content

Cells were seeded at a density of 8000 cells per well into 96-well transparent cell-culture plates and incubated overnight at 37 °C. The following day, the cultures were transferred to serum-free conditions and the cells were further incubated for 24 h. Treatment with 10 µM losartan (Sigma-Aldrich, USA) or 10 µM FPS-ZM1 (Merck Millipore, USA) started 24 h or 30 min prior to the stimulation, respectively. siRNA for RAGE or AT1a was performed 24 h prior to the stimulation. Thereafter, the cells were treated for 1 h with DMEM without phenol, 1 mM IBMX, and 1 µM Forskolin including each reagent at the indicated concentration at 37 °C, 5% CO2. Triton X was then added at a final concentration of 1%, and cell lysates were prepared after shaking the plates for 30 min. Finally, the cell lysates were transferred to 384-well white plates and cAMP levels were measured using the cAMP dynamic 2 kit (Cisbio, France). The emission signals were measured at 620 and 665 nm after excitation at 320 nm, using the ARTEMIS plate reader(Furuno Electric Co. Ltd, Japan).

### siRNA

Silencer Select siRNA for RAGE (s135582 and s135583) or AT1a (s127417) (Thermo Fisher Scientific, USA) was transfected into NRK52E cells plated the previous day in a serum- and antibiotic-free medium using lipofectamine RNAiMAX (Thermo Fisher Scientific, USA), according to the manufacturer’s instructions. The cells were treated with BSA or AGE-BSA 24 h post-transfection.

### Quantitative real-time PCR

Total RNA was extracted using the RNeasy Mini Kit (Qiagen, Germany) and transcribed into cDNA using the ReverTraAce qPCR RT kit (TOYOBO, Japan). Quantitative real-time PCR was performed with a model 7900 sequence detector system (Life Technologies, USA) using TaqMan gene-expression assays for *AT1a* (Rn02758772_s1 with the reference sequence in NM_030985.4) (Life Technologies, USA). We also performed SYBR Green real-time PCR for *RAGE* (5′-TGAAGGTGGAACAGTCGCTC-3′ and 5′-ATCACCGGTTTCTGTGACCC-3′) and *GAPDH* (5′-GCCATCAATGACCCCTTCATT-3′ and 5′-TCTCGCTCCTGGAAGATGG-3′). *GAPDH* was used as an internal control to normalize the expression level of each gene using the ΔΔCt method.

### Luciferase reporter assay

NRK-52E cells seeded in a 24-well plate were transfected with a plasmid containing luciferase driven by the NF-κB binding site (Promega, USA). The cells were cultured in medium containing 0.1% FBS for 24 h with or without an ARB, olmesartan (Sigma-Aldrich, USA). The cells were then pre-treated with or without a RAGE inhibitor, FPS-ZM1 (Merck Millipore, USA), for 30 min and stimulated with 50 μg/mL BSA (Merck Millipore, USA) or 50 μg/mL AGE-BSA (Biovison, USA) for 2 h. Luciferase activity was measured using the luciferase reporter assay system (Promega, USA), according to the manufacturer’s protocol. Measured values were normalized according to the protein concentration in each well.

### Epithelial–mesenchymal transition

NRK-52E cells were pre-treated overnight with or without irbesartan or losartan (Sigma-Aldrich, USA), and subsequently stimulated with 100 µg/mL BSA (Merck Millipore, USA), 100 µg/mL AGE-BSA (Biovision, USA), 3 µg/mL bovine HMGB1 (Iwai, Japan), or 20 ng/mL TGFβ (Wako, Japan) for 72 h in media supplemented with 0.1% FBS.

### Statistics

All data are presented as the mean ± SEM. The statistical difference between two treatments or among multiple treatments was determined using the Student’s *t*-test or one-way ANOVA with Bonferroni testing, respectively.

## Results

### RAGE formed a complex with AT1 on the cell membrane

**Figure 1:**
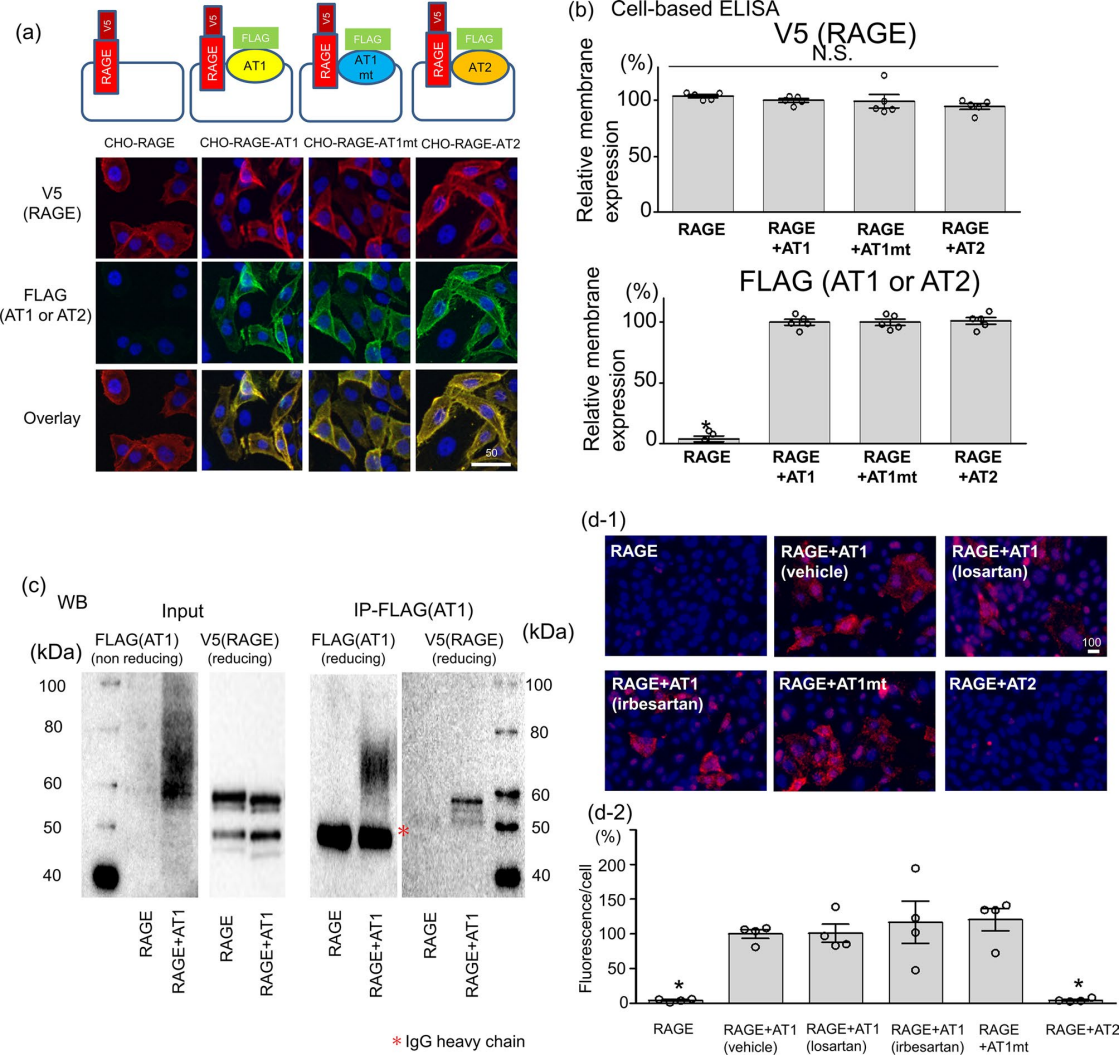
Physical interaction between RAGE and AT1 in transgenic CHO cells. (**a**) Immunofluorescence of CHO cells stably expressing V5-tagged RAGE alone or V5-tagged RAGE with FLAG-tagged AT1, FLAG-tagged AT1 mutant (AT1mt), or FLAG-tagged AT2 using anti-V5 or anti-FLAG antibody. Scale bar (μm) The red image indicates positive RAGE on CHO cells and the green images indicates positive AT1/AT1mt/AT2 on CHO cells. (**b**) Quantification of membrane-expressing V5-tagged RAGE, and FLAG-tagged AT1, AT1 mutant or AT2 by cell-based ELISA. Absorbance in CHO-RAGE-AT1 was normalized to 100% (number(n) = 5, each). *p < 0.01 vs CHO-RAGE-AT1. (**c**) Immunoprecipitation of membrane proteins with Anti-FLAG antibody followed by immunoblotting with Anti-FLAG antibody or anti-V5 antibody. Full-length blots are presented in Supplementary Fig. 1(d-1,2) In situ PLA performed under non-permeabilized condition using antibodies for mouse anti-V5 antibody and rabbit anti-FLAG antibody. Treatment with 10 μM losartan (AT1 blocker, ARB) or 10 μM irbesartan (ARB) was carried out for 24 h. The red fluorescence indicates the membrane proximity of V5-tagged RAGE and FLAG-tagged AT1/AT1mt/AT2 (< 30–40 nm). The graph indicates fluorescence/number of nuclei (% of that in CHO-RAGE-AT1 with vehicle treatment) (n = 4, each). Scale bar (μm); *p < 0.01 vs CHO-RAGE-AT1 with vehicle treatment. The differences were determined by one-way ANOVA with Bonferroni correction.

**Figure 2:**
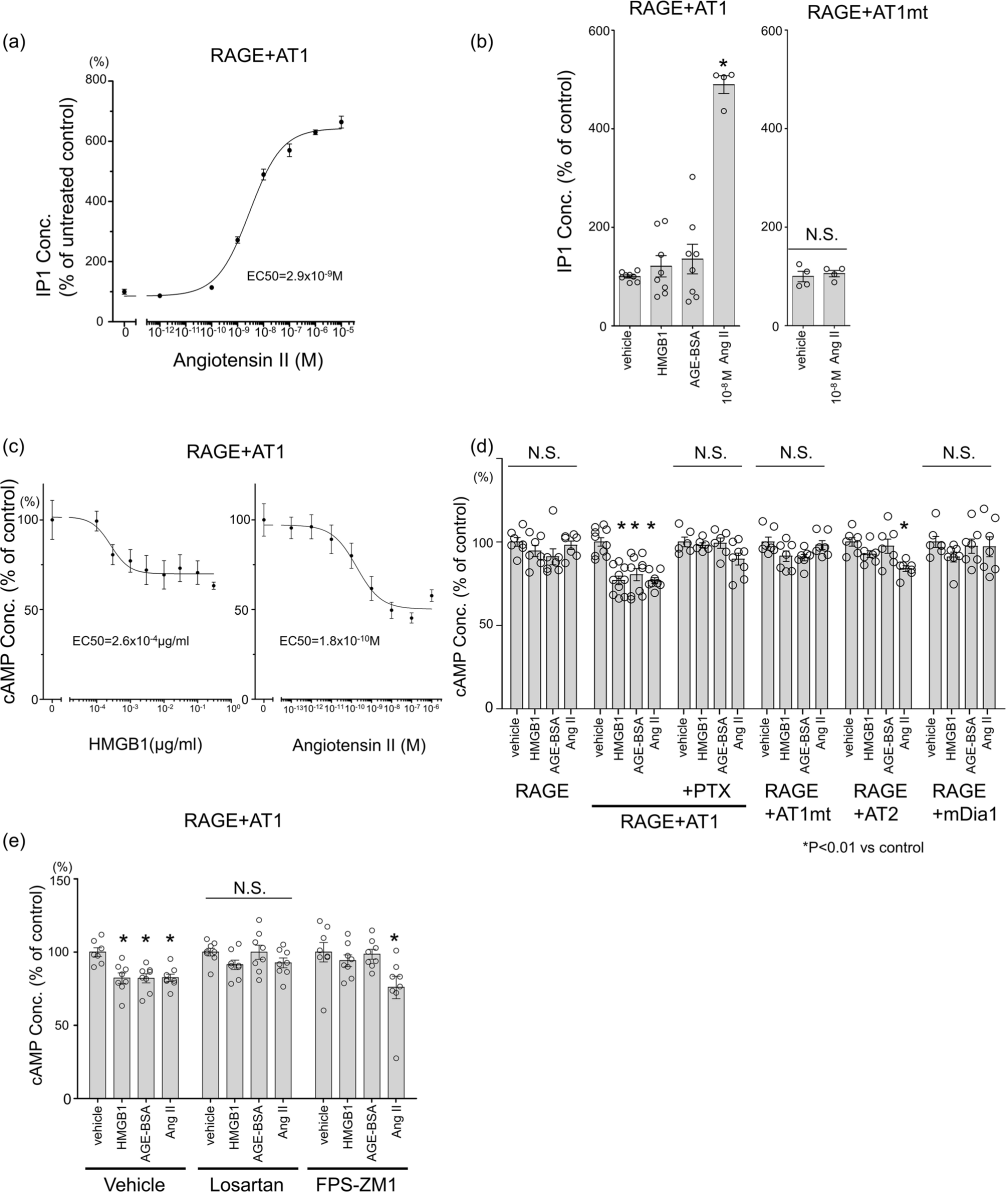
G protein-selective AT1 activation by RAGE ligands in transgenic CHO cells. (**a**) Dose-dependent response in inositol monophosphate (IP1) concentration in response to angiotensin II (10^–12^–10^–5^ M) in CHO-RAGE-AT1. The measurement is expressed as % of IP1 concentration in the vehicle treatment. (number(n) = 4, each). (**b**) IP1 concentration in response to vehicle, 0.3 µg/mL HMGB1, 100 µg/mL AGE-BSA, or 10^–10^M angiotensin II in genetically engineered CHO cells. The concentration in the control wells of each cell type was normalized to 100% (n = 8 for vehicle, HMGB-1, and AGE-BSA, and n = 4 for angiotensin II in CHO-RAGE-AT1, n = 4 in CHO-RAGE-AT1mt) *p < 0.01 vs. vehicle treatment. (**c**) Dose-dependent response in cyclic adenosine monophosphate (cAMP) concentration in response to HMGB1(10^–4^–10^1^ µg/ml) or angiotensin II (10^–12^-10^–6^ M) in CHO-RAGE-AT1. Cells were treated with 1 µM forskolin to induce cAMP accumulation. n = 4, each). (**d**) cAMP concentration in response to vehicle ,0.3 µg/mL HMGB1, 100 µg/mL AGE-BSA, or 10^–10^ M angiotensin II in genetically engineered CHO cells. Gi inhibitor, pertussis toxin (PTX), was pre-treated at 25 ng/mL for 12 h before stimulation. The concentration in control-treated wells in each cell type was normalized to 100% (n = 10 in CHO-RAGE-AT1, and n = 7 in CHO-RAGE, CHO-RAGE-AT1 with PTX treatment, CHO-RAGE-At1mt, RAGE-AT2, and RAGE-mDia-1) *p < 0.01 vs. vehicle treatment. (**e**) cAMP concentration in response to vehicle, 0.3 µg/mL HMGB1, 100 µg/mL AGE-BSA, or 10^–10^ M angiotensin II in CHO-RAGE-AT1 pre-treated with vehicle, 10 µM losartan (AT1 blocker), or 10 µM FPS-ZM1 (RAGE inhibitor) (n = 8, each). *p < 0.01 vs. vehicle treatment. The differences were determined by one-way ANOVA with Bonferroni correction.

To understand the signaling mechanisms triggered by RAGE stimulation, we generated CHO cells that express no endogenous AT1 stably encoding V5-tagged RAGE alone, and V5-tagged RAGE with FLAG-tagged AT1, FLAG-tagged mutated AT1 that impairs the ability to activate G protein (AT1mt)^16,17^, or FLAG-tagged AT2–the AT1 isoform (CHO-RAGE, CHO-RAGE-AT1, CHO-RAGE-AT1mt, and CHO-RAGE-AT2, respectively). The immunofluorescence images of these cell lines are shown in Fig. 1a. The similar expression levels of the corresponding receptors on the cell surface were confirmed by cell-based ELISA under non-permeabilized condition (Fig. 1b). The immunoprecipitation of membrane proteins with an anti-FLAG antibody revealed that RAGE is immunoprecipitated with AT1 (Fig. 1c). An in situ PLA assay conducted under non-permeabilized conditions to determine the membrane proximity of two molecules (< 30–40 nm) exhibited a similar enhancement of red fluorescence in CHO-RAGE-AT1 and CHO-RAGE-AT1mt, but not in CHO-RAGE-AT2, implying the specific proximity of RAGE and AT1 either with or without mutation (Fig. 1d-1,2). These results are consistent with a previous study that demonstrated the oligomerization between AT1 and RAGE using the bioluminescence resonance energy transfer method^15^. Losartan and irbesartan did not alter the proximity between RAGE and AT1, indicating that the heterodimerized RAGE/AT1 complex is not uncoupled by ARBs (Fig. 1d-1,2).

### RAGE ligands induced G protein-selective AT1 activation

AngII binds to AT1 to initiate intracellular signaling cascades by activating specific G proteins α subunit, including Gαq/11 (Gq), Gαi/0 (Gi), and Gα12/13^12^. Herein, we assessed the activation of Gq and Gi by measuring inositol monophosphate (IP1) accumulation and the inhibition of forskolin-induced cyclic adenosine monophosphate (cAMP) production, respectively. AngII increased inositol monophosphate (IP1) levels in CHO-RAGE-AT1, but not in CHO-RAGE-AT1mt (Fig. 2a, b). However, the RAGE ligands HMGB1 and AGE-BSA did not alter IP1 levels in CHO-RAGE-AT1, suggesting that RAGE ligands cannot activate Gq signaling (Fig. 2b). By contrast, the treatment of CHO-RAGE-AT1 with HMGB1 as well as AngII decreased forskolin-induced increase in cAMP levels in a dose-dependent manner (Fig. 2c). The effect of AngII or RAGE ligands on cAMP levels was absent in CHO-RAGE, and was abrogated upon treatment with a Gi inhibitor, pertussis toxin, in CHO-RAGE-AT1 (Fig. 2d). The treatment of CHO-RAGE-AT1mt with RAGE ligands did not affect cAMP levels, supporting the dependence of this phenomenon on the AT1-G protein pathway (Fig. 2d). While the treatment of CHO-RAGE-AT2 with RAGE ligands did not affect cAMP levels, Ang II reduced cAMP levels consistent with previous studies that AngII-AT2 activates Gi (Fig. 2d)^18^. Notably, mDia1 overexpression in CHO-RAGE cells did not affect RAGE ligand-mediated cAMP reduction, indicating that AT1 is essential for the RAGE-mediated activation of G proteins (Fig. 2d). Additionally, the treatment of cells with an ARB, losartan, inhibited both RAGE ligand- and AngII-induced reduction of cAMP levels (Fig. 2e). The treatment of cells with a RAGE inhibitor, FPS-ZM1 abrogated RAGE ligand-induced reduction of cAMP levels, but not AngII-induced reduction of cAMP levels (Fig. 2e). These data indicated that RAGE ligands selectively activate the G protein pathways mediated by AT1, and that this activation is inhibited by the pharmacological blockade of AT1.

### AGE-induced cellular signaling was blocked by the inhibition or knockdown of AT1 in kidney epithelial cells

**Figure 3:**
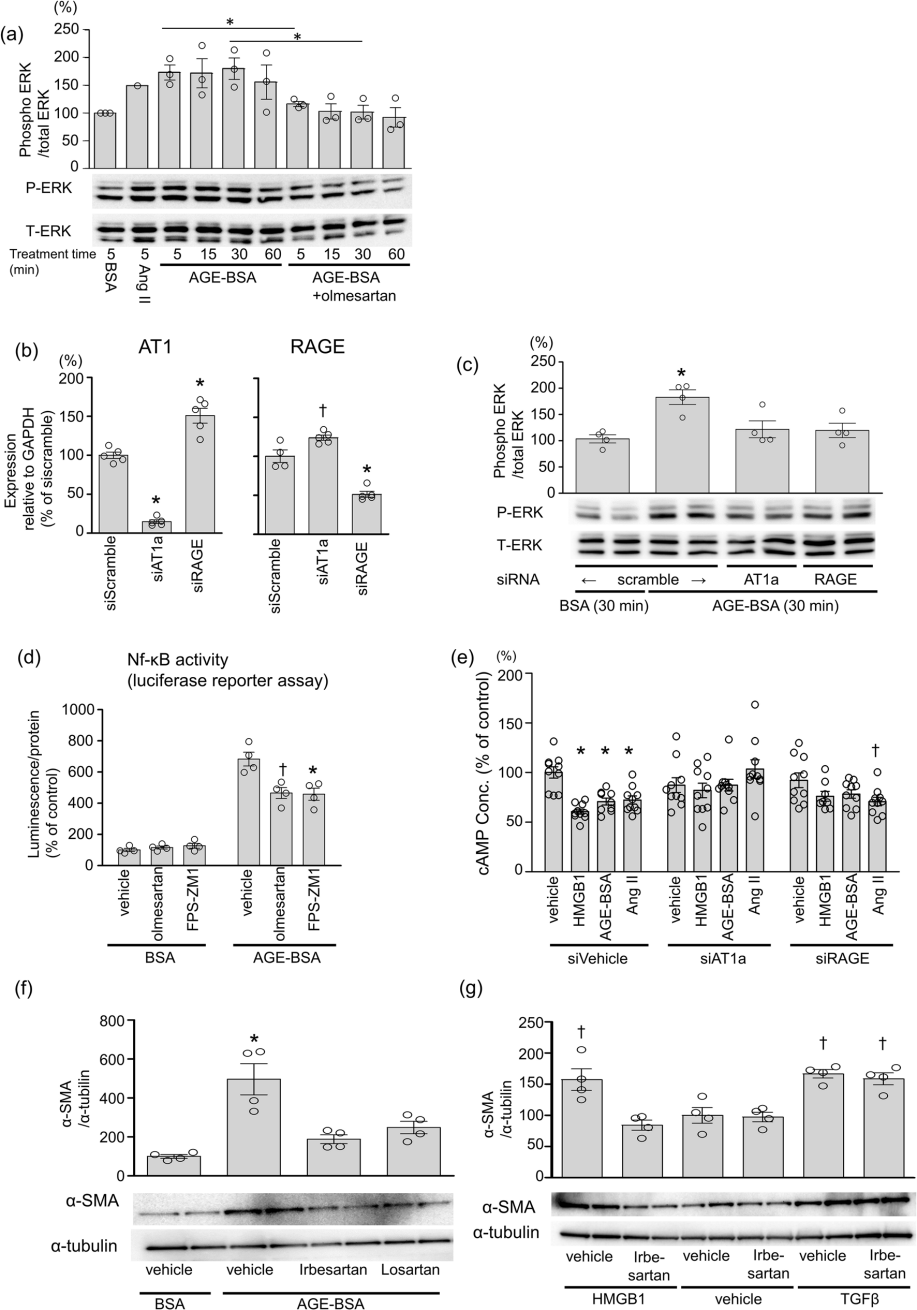
AGE-BSA induces the AT1-dependent signaling cascade in rat kidney epithelial cells. (**a**) Immunoblot analysis of phosphorylation of ERK in response to 100 µg/mL BSA, Ang II or AGE-BSA for 5, 15,30, or 60 min with or without 1 µM olmesartan (AT1 blocker, ARB) in NRK52E cells. The graph indicates the intensity ratio of phosphorylated ERK (P-ERK) /total ERK(T-ERK) normalized to that with vehicle control (number(n) = 1, Ang II, n = 4, the other groups). *p < 0.05 determined by Student’s *t*-test. (**b**) Confirmation of knockdown efficiency of siRNA by quantitative real-time PCR in NRK-52E. (n = 5, each) The expression level of each gene was normalized using GAPDH mRNA as an internal control. The differences were determined by one-way ANOVA with Bonferroni correction. *p < 0.01 vs scramble siRNA; ♰*p* < 0.05 vs scramble siRNA. (**c**) Immunoblot analysis of phosphorylation of ERK in response to 100 µg/mL BSA or AGE-BSA for 30 min in combination with siRNA of scramble, RAGE or AT1 in NRK52E cells. The graph indicates the intensity ratio of P-ERK /T-ERK normalized to that with vehicle control (n = 4, each). The differences were determined by one-way ANOVA with Bonferroni correction. *p < 0.01 vs. scramble siRNA treated with BSA. (**d**) NF-κB luciferase reporter assay performed employing a treatment regimen of 50 μg/mL BSA or AGE-BSA for 2 h on treatment with 1 μM olmesartan (ARB) or 1 μM FPS-ZM1 (RAGE inhibitor) (n = 4, each). The differences were determined by one-way ANOVA with Bonferroni correction. *p < 0.01 vs. AGE-BSA treatment; ♰*p* < 0.05 vs. AGE-BSA treatment. (**e**) cAMP concentration in response to vehicle ,0.3 µg/mL HMGB1, 100 µg/mL AGE-BSA, or 10^–10^ M angiotensin II after the treatment with the indicated siRNA in NRK52E (n = 10, each). The concentration in vehicle-treated wells in cells with siVehicle was normalized to 100%. The differences were determined by one-way ANOVA with Bonferroni correction. *p < 0.01 vs. vehicle treatment; ♰*p* < 0.05 vs. vehicle treatment. (**f**, **g**) Immunoblot analysis of α-smooth muscle actin (SMA) as a measure of epithelial–mesenchymal transition after stimulation with (**f**) 100 μg/mL BSA, 100 μg/mL AGE-BSA, (**g**) vehicle, 3 µg/mL HMGB1, or 20 ng/mL TGFβ for 72 h. (n = 4, each). Treatment with 1 μM losartan (ARB), or irbesartan (ARB) was carried out 24 h before stimulation. The graph indicates the intensity ratio of α-SMA /control α-tubulin normalized to that with BSA (f) or vehicle (**g**). The differences were determined by one-way ANOVA with Bonferroni correction. *p < 0.05 vs. the other treatments; ♰*p* < 0.05 vs. vehicle treatment. Full-length blots are presented in Supplementary Fig. 2.

We then used NRK52E cells, rat renal proximal tubular epithelial cells, to clarify if the observed machinery is relevant in primary cells that express endogenous AT1 and RAGE. AGE-BSA increased ERK1/2 phosphorylation, and the increase at 5 or 30 min was inhibited by the treatment of olmesartan (Fig. 3a). siRNA-mediated AT1 knockdown or the treatment of losartan significantly inhibited AGE-induced ERK 1/2 phosphorylation as well as RAGE knockdown (Fig. 3b, c). AGE-induced inflammatory response assessed by NF-κB reporter assay was inhibited similarly by an ARB, olmesartan and a RAGE inhibitor, FPS-ZM1 (Fig. 3d). HMGB1 and AGE-BSA activated the Gi-dependent cell signaling as well as AngII as shown by the inhibition of forskolin-induced cAMP production (Fig. 3e). Either siRNA of AT1 or RAGE abolished the effect of these RAGE ligands on the forskolin-induced cAMP production (Fig. 3e).

### ARBs abolished RAGE ligand-induced renal epithelial–mesenchymal transition

We used the immunoblot analysis of α-smooth muscle actin (SMA), a molecular marker of mesenchymal cells^19^ to further investigate whether RAGE ligands cause AT1-dependent Epithelial–mesenchymal transition (EMT) (Fig. 3f). Treatment of NRK52E cells with AGE-BSA for 72 h increased α-SMA and the treatment with ARBs (irbesartan and losartan) attenuated the AGE-induced EMT. Treatment with HMGB1 also induced EMT and ARB was shown to abrogate HMGB1-induced EMT. ARB treatment did not attenuate EMT induced by Transforming Growth Factor-β (TGF-β) (Fig. 3g).

## Discussion

Previous studies have reported the interaction between RAGE and the RAS primarily at the transcriptional level^20–23^. The blockades of RAS downregulate TNFα-induced RAGE expression^20^ and attenuate RAGE-mediated signaling pathways such as AGE-induced NADPH oxidase signaling^22^. Pickering et al. recently reported that a heteromeric complex is formed between AT1 and RAGE, and that AngII transactivates RAGE to initiate RAGE-mediated signaling^15^. In the present study, we reported that the physical interaction between RAGE and AT1 on the cell membrane facilitates the signal transduction pathway from ligand binding with RAGE to the activation of AT1. These findings suggest that the crosstalk between RAGE and AT1 is bidirectional and far more direct than previously supposed.

Notably, our data suggest that mDia1 has no effect on RAGE ligand-mediated signaling in CHO cells lacking AT1. mDia1 is a key component in RAGE signaling due to its interaction with the RAGE cytoplasmic domain^4,5^. Hence, further studies are required to understand the role of mDia1 in RAGE signaling with emphasis on its interaction with AT1. Importantly, our data suggest that AT1 activation induced upon the binding of RAGE ligands to RAGE has distinct characteristics from that induced upon AngII binding. AngII activates Gi and Gq through AT1; conversely, Gi, but not Gq, is activated by RAGE ligands via the AT1/RAGE complex. This can be explained by the recently proposed machinery of biased AT1 activation. Recent structural analyses have revealed that β-arrestin-biased agonists induce a less “open’’ conformational change of AT1 compared to that induced by Ang II or other agonists with enhanced Gq coupling activity^13,14^. It was also shown that β-arrestin-biased AngII analogs preferentially activate Gi over Gq^12^. Future structural analysis will be required to clarify how the ligand–RAGE interaction induces conformational change of AT1, but the mechanism of biased activation may explain why RAGE ligands and AngII exert distinct physiological and pathophysiological effects. For example, RAGE ligands do not increase blood pressure as much as AngII because the pressor function of AngII relies on Gq-dependent AT1 signaling. Among the G protein activation induced by AngII binding to AT1, we did not assess the G12/13 pathway that stimulates the activation of Rho kinases. It was reported that biased AngII analogs preferentially activate G12/13 as well as Gi over Gq^12^. Further studies are required to clarify if RAGE ligands activate G12/13 via an AT1-dependent fashion.

We found that the pharmacological and genetic inhibition of AT1 abolished RAGE ligand-induced cellular responses in renal tubular epithelial cells. ARBs were shown to attenuate RAGE ligand-induced EMT, a crucial cellular response in the development of diabetic nephropathy^24,25^. While the protective role of ARBs in the development of diabetic nephropathy has been extensively studied, certain inherent mechanisms have been presumed to exist that modulate the direct inhibition of Ang II-mediated AT1 activation. Our current findings provide a novel molecular basis to support the role of ARBs in diabetic nephropathy by directly inhibiting the signaling pathway of RAGE. Interestingly, in line with our findings, it was reported that an ARB, valsartan, inhibited the renal injury induced by the infusion of AGE-modified rat serum albumin, while the AGE inhibitor pyridoxamine inhibited the renal injury induced by Ang II infusion^26^. Nevertheless, given that ACE inhibitors are also shown to prevent the development of diabetic nephropathy, further investigation will be required to elucidate if, and to what extent, the pharmacological inhibition of AT1 has an additive class effect on the inhibition of diabetic complications other than the blockade of Ang II signaling.

In conclusion, the present study demonstrates that the cell-surface complex of RAGE and AT1 mediates the selective activation of AT1 induced by RAGE ligands. This phenomenon is relevant in RAGE ligand-induced intracellular signal transduction and cell phenotype change in kidney proximal tubular cells. These findings provide novel insight into the crosstalk between RAGE and AT1, both of which have critical roles in the development of cardiovascular diseases.

## Supplementary Information

Supplementary Figures
