# Supplementary material for: RAGE ligands stimulate angiotensin II type I receptor (AT1) via RAGE/AT1 complex on the cell membrane
Source: Sci Rep. 2021 Mar 11;11:5759. doi: 10.1038/s41598-021-85312-4 (PMC7952713; doi:10.1038/s41598-021-85312-4)
Supplement: Supplementary file 1 — Supplementary Figures [file 41598_2021_85312_MOESM1_ESM.pptx]

## Slide 1
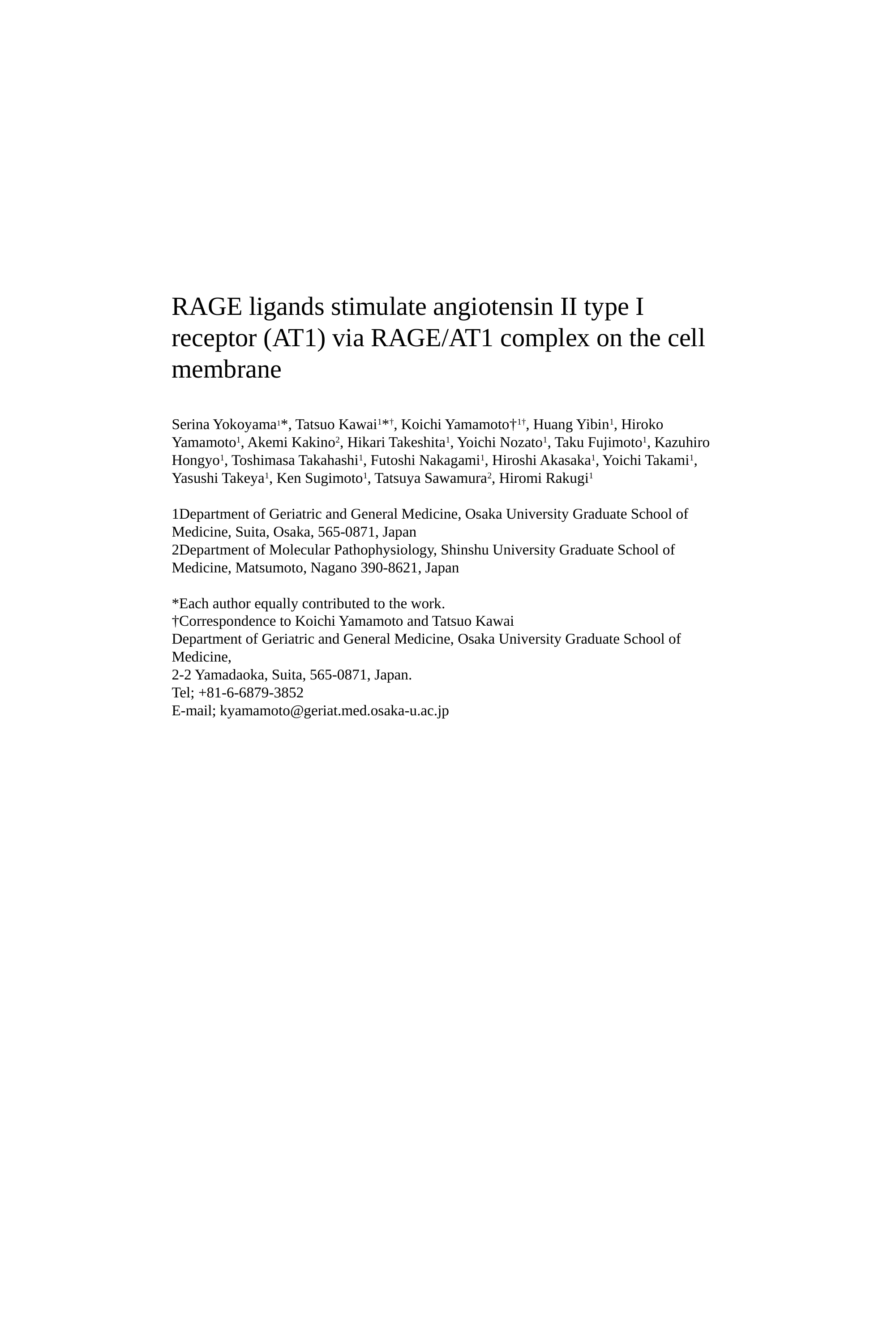

RAGE ligands stimulate angiotensin II type I receptor (AT1) via RAGE/AT1 complex on the cell membrane
Serina Yokoyama1*, Tatsuo Kawai1*†, Koichi Yamamoto†1†, Huang Yibin1, Hiroko Yamamoto1, Akemi Kakino2, Hikari Takeshita1, Yoichi Nozato1, Taku Fujimoto1, Kazuhiro Hongyo1, Toshimasa Takahashi1, Futoshi Nakagami1, Hiroshi Akasaka1, Yoichi Takami1, Yasushi Takeya1, Ken Sugimoto1, Tatsuya Sawamura2, Hiromi Rakugi1
1Department of Geriatric and General Medicine, Osaka University Graduate School of Medicine, Suita, Osaka, 565-0871, Japan
2Department of Molecular Pathophysiology, Shinshu University Graduate School of Medicine, Matsumoto, Nagano 390-8621, Japan
*Each author equally contributed to the work.
†Correspondence to Koichi Yamamoto and Tatsuo Kawai
Department of Geriatric and General Medicine, Osaka University Graduate School of Medicine,
2-2 Yamadaoka, Suita, 565-0871, Japan.
Tel; +81-6-6879-3852
E-mail; kyamamoto@geriat.med.osaka-u.ac.jp

## Slide 2
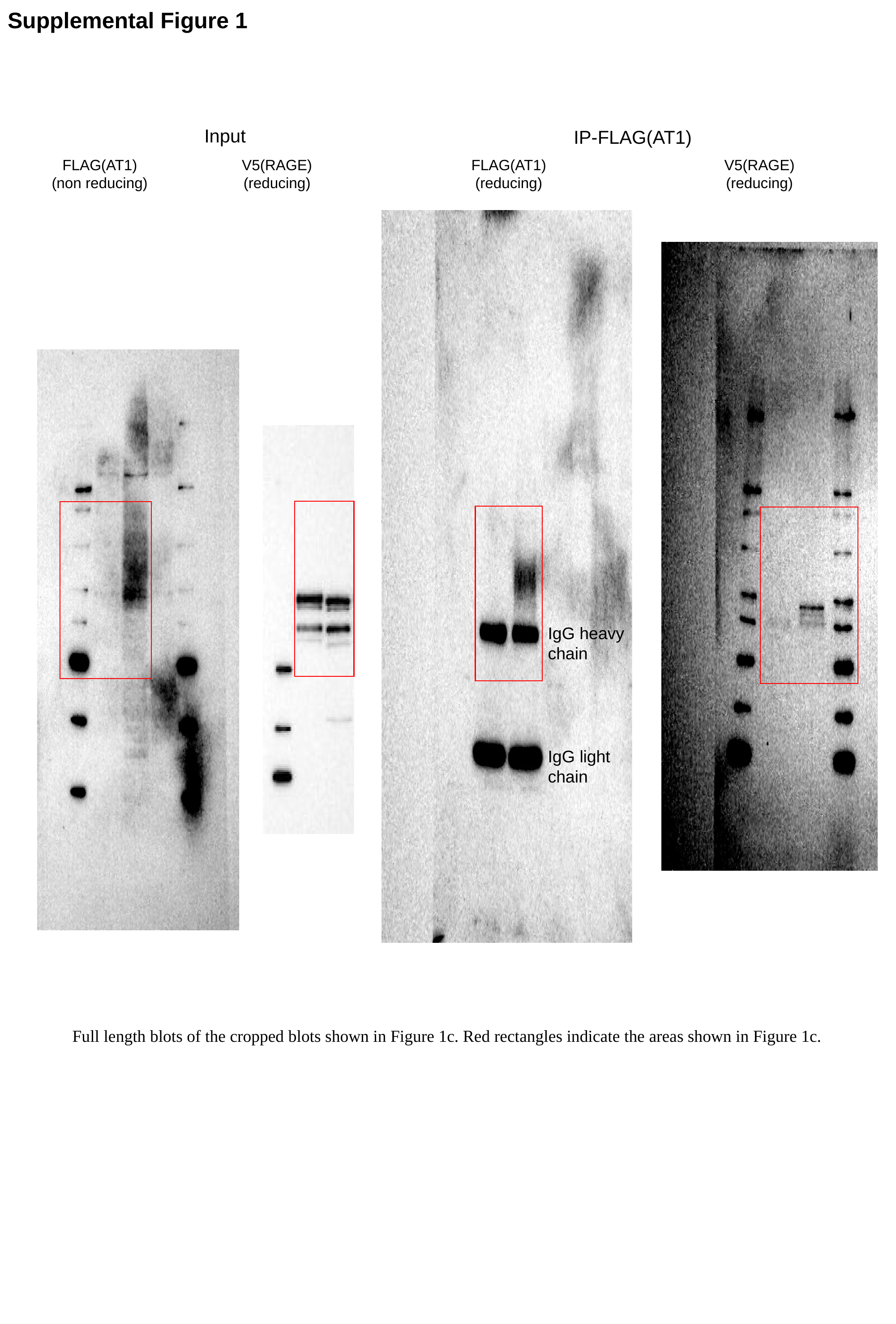

Supplemental Figure 1
Input
IP-FLAG(AT1)
FLAG(AT1)
(non reducing)
V5(RAGE)
(reducing)
FLAG(AT1)
(reducing)
V5(RAGE)
(reducing)
IgG heavy
chain
IgG light
chain
Full length blots of the cropped blots shown in Figure 1c. Red rectangles indicate the areas shown in Figure 1c.

## Slide 3
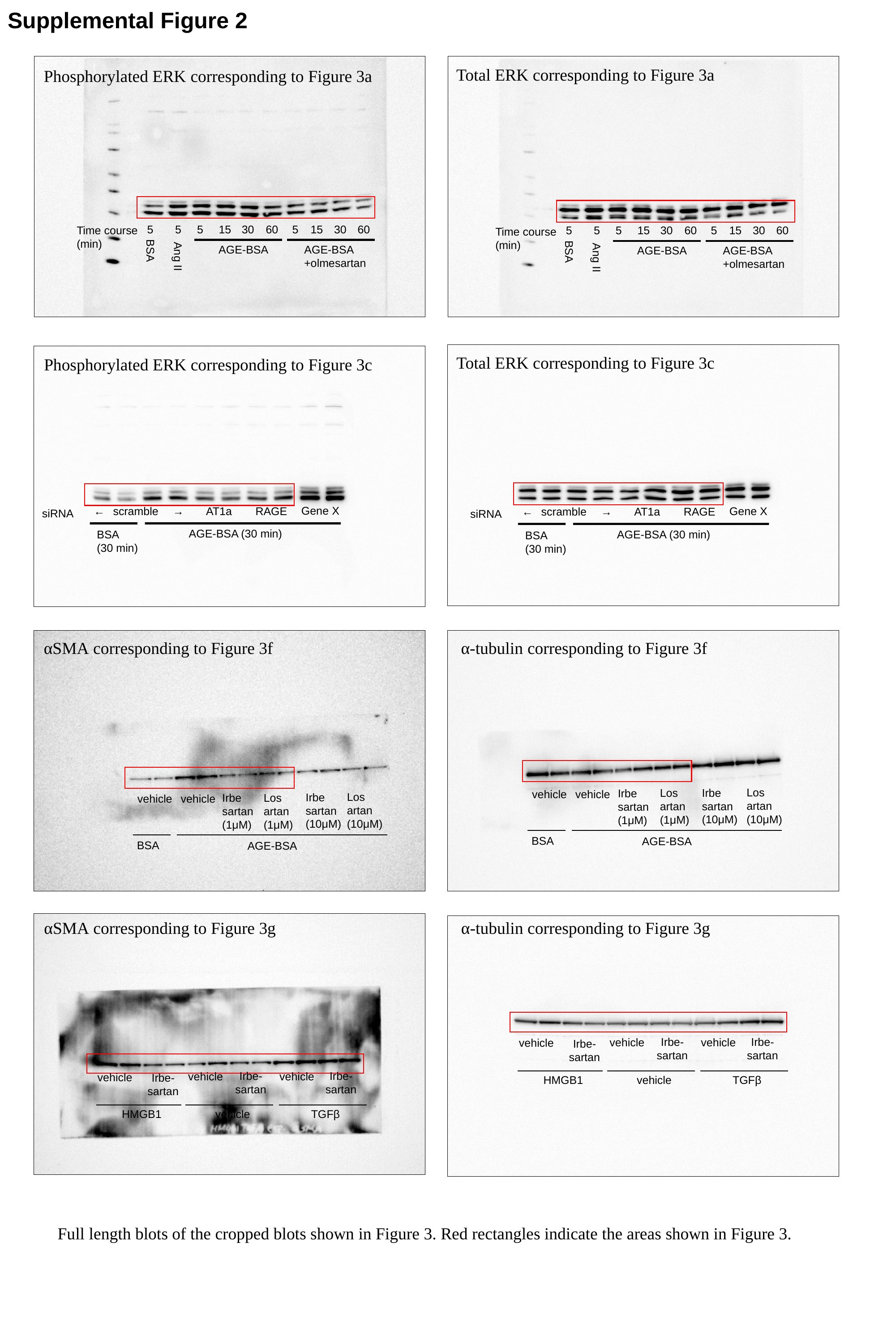

Supplemental Figure 2
Total ERK corresponding to Figure 3a
Phosphorylated ERK corresponding to Figure 3a
5
5
5
15
30
60
5
15
30
60
Time course
(min)
5
5
5
15
30
60
5
15
30
60
Time course
(min)
AGE-BSA
AGE-BSA
+olmesartan
AGE-BSA
AGE-BSA
+olmesartan
BSA
BSA
Ang II
Ang II
Total ERK corresponding to Figure 3c
Phosphorylated ERK corresponding to Figure 3c
Gene X
←
scramble
→
AT1a
RAGE
Gene X
←
scramble
→
AT1a
RAGE
siRNA
siRNA
AGE-BSA (30 min)
AGE-BSA (30 min)
BSA
(30 min)
BSA
(30 min)
αSMA corresponding to Figure 3f
α-tubulin corresponding to Figure 3f
Los
artan
(10μM)
Irbe
sartan
(10μM)
Los
artan
(1μM)
Irbe
sartan
(1μM)
vehicle
vehicle
Los
artan
(10μM)
Irbe
sartan
(10μM)
Los
artan
(1μM)
Irbe
sartan
(1μM)
vehicle
vehicle
BSA
AGE-BSA
BSA
AGE-BSA
αSMA corresponding to Figure 3g
α-tubulin corresponding to Figure 3g
Irbe-
sartan
Irbe-
sartan
vehicle
vehicle
vehicle
Irbe-
sartan
Irbe-
sartan
Irbe-
sartan
vehicle
vehicle
vehicle
Irbe-
sartan
HMGB1
vehicle
TGFβ
HMGB1
vehicle
TGFβ
Full length blots of the cropped blots shown in Figure 3. Red rectangles indicate the areas shown in Figure 3.
